# Discovery of *bla*_OXA-199_, a Chromosome-Based *bla*_OXA-48_-Like Variant, in *Shewanella xiamenensis*

**DOI:** 10.1371/journal.pone.0048280

**Published:** 2012-10-24

**Authors:** Zhiyong Zong

**Affiliations:** 1 Center of Infectious Diseases, West China Hospital, Sichuan University, Chengdu, China; 2 Division of Infectious Diseases, The State Key Laboratory of Biotherapy, Chengdu, China; Belgian Nuclear Research Centre SCK/CEN, Belgium

## Abstract

**Introduction:**

*bla*_OXA-48_ is a globally emerging carbapenemase-encoding gene. The progenitor of *bla*_OXA-48_ appears to be a *Shewanella* species. The presence of the *bla*_OXA-48_-like gene was investigated for two *Shewanella xiamenensis* strains.

**Methods:**

Strain WCJ25 was recovered from post-surgical abdominal drainages, while S4 was the type strain of *S. xiamenensis*. Species identification for WCJ25 was established by sequencing the 16S rDNA and *gyrB* genes. PCR was used to screen the *bla*_OXA-48_-like genes and to obtain their complete sequences. A phylogenetic tree of the *bla*_OXA-48_-like genes was constructed. The genetic context of the *bla*_OXA-48_-like gene in strain WCJ25 was investigated by inverse PCR using self-ligated AseI- or RsaI-restricted WCJ25 DNA fragments as template, while that in strain S4 was determined by PCR mapping using that in WCJ25 as template.

**Results:**

A new *bla*_OXA-48_ variant, designated *bla*_OXA-48b_, with four silent nucleotide differences from the *bla*_OXA-48_ (designated *bla*_OXA-48a_) found in the *Enterobacteriaceae* was identified in strain S4. Strain WCJ25 had a new *bla*_OXA-48_-like variant, *bla*_OXA-199_, with five nucleotide differences from *bla*_OXA-48_a and *bla*_OXA-48b_. The OXA-199 protein has three amino acid substitutions (H37Y, V44A and D153G) compared with OXA-48. Both *bla*_OXA-48b_ and *bla*_OXA-199_ were found adjacent to genes encoding a peptidase (indicated as orf), a protein of unknown function (*sprT*), an endonuclease I (*endA*), and a ribosomal RNA methyl transferase (*rsmE*) upstream and to transcriptional regulator gene *lysR* and an acetyl-CoA carboxylase-encoding gene downstream. In addition, the insertion sequence IS*Shes2* was found inserted downstream of *bla*_OXA-199_ but not of *bla*_OXA-48b_. The 26 bp sequences upstream and 63 bp downstream of *bla*_OXA-48a_, *bla*_OXA-48b_ and *bla*_OXA-199_ were identical.

**Conclusions:**

*bla*_OXA-48a_, *bla*_OXA-48b_ and *bla*_OXA-199_ might have a common origin, suggesting that the *bla*_OXA-48a_ gene found in the *Enterobacteriaceae* could have originated from the chromosome of *S. xiamenensis*.

## Introduction

*bla*_OXA-48_, encoding the carbapenem-hydrolyzing enzyme OXA-48, was initially found in *Klebsiella pneumoniae* from Turkey [1] and has now been spread to other *Enterobacteriaceae* species in a few countries [2], [3]. Several *bla*_OXA-48_-like variants have been identified recently, including *bla*_OXA-162_ (GenBank Accession no. GU197550; one nucleotide different from *bla*_OXA-48_), *bla*_OXA-163_ (98.1% nucleotide identity with *bla*_OXA-48_) [4], *bla*_OXA-181_ (94.4% nucleotide identity with *bla*_OXA-48_) [5], *bla*_OXA-204_ (nucleotide sequence not available but encoding two amino acid substitutions compared with OXA-48) and *bla*_OXA-232_ (nucleotide sequence not available but encoding a single amino acid substitution compared with OXA-181) [3]. The *bla*_OXA-48_ gene was previously proposed as been derived from the chromosome-encoded *bla*_OXA-54_ of *Shewanella oneidensis*, but the two genes have only 84% nucleotide identity [6]. Through analyzing the complete genome sequences of a few strains belonging to various *Shewanella* species available in the GenBank, the *bla*_OXA-48_-like genes are present on the chromosome of several *Shewanella* species with at least 80% identity to *bla*_OXA-48_. Thus, the actual progenitor of *bla*_OXA-48_ may rather lie within a *Shewanella* species other than *S. oneidensis*. A *Shewanella* clinical strain previously isolated and characterized [7] and the type strain of *Shewanella xiamenensis* [8] were investigated for the presence of a *bla*_OXA-48_-like gene.

## Methods

### Strains

*Shewanella* isolate WCJ25 was recovered from post-surgical abdominal drainages of a patient with pancreatitis and was identified as *S. xiamenensis* based on the close identity (99.6% for 16S rDNA gene and 98.5% for *gyrB*) between WCJ25 and the *S. xiamenensis* type strain S4 [7]. The *S. xiamenensis* type strain S4 was provided by Prof. Zhang Xiaobo, Zhejiang University.

### Screening for *bla*_OXA-48_-like Genes

PCR was used to screen *bla*_OXA-48_-like genes and to obtain the complete sequence of the *bla*_OXA-48_-like gene with primers listed in Table 1. PCR was conducted using the ExTaq mix (Takara, Dalian, China) with the conditions being 94°C for 5 min, 30 cycles (94°C for 30s, 52°C for 45 s, 72°C for 1 min) and a final elongation step at 72°C for 7 min. The amplicons were purified using the OMEGA Cycle Pure kit (Norcross, GA, USA) and sequenced.

**Table 1:** Primers used.

Primer	Sequence 5′-3′	Target	Source
OXA48/54IF	AGCAAGGATTTACCAATAAT	*bla*_OXA-48_-like genes,	Valenzuela JK
OXA48/54IR	GGCATATCCATATTCATC	screening	unpublished
OXA48-up1	ATTAAGCAAGGGGACGTTATG	*bla*_OXA-48_-like genes,	This study
OXA48-dw1	GAGCATCAGCATTTTGTCCA	complete sequences	This study
OXA48-IR2	GCAACTACGCCCTGTGATTT	*bla*_OXA-48_-like genes	This study
OXA48-dw2	GTTAGCGCGTATTTGTGTG	Downstream of *bla*_OXA-199_	This study
OXA199-up1	TAAGCCTGAACGCCCTAGAA	Upstream of *bla*_OXA-199_	This study
tnpA_Shewa-R1	AATAGTTTCGGCAGGGGTTT	*tnpA* of IS*Shes2*	This study
orfJ25-R1	ACGGCTAATGGTTGAGGTTG	*rsmE*	This study
Orf25-R2	CCGTCATAGCGATTTCTTCC	*rsmE*	This study
aceCoA-R2	TTGGGCAATAAAGCCGATAC	*acc*	This study

### Phylogenetic Analysis of the *bla*_OXA-48_-like Genes

**Figure 1:**
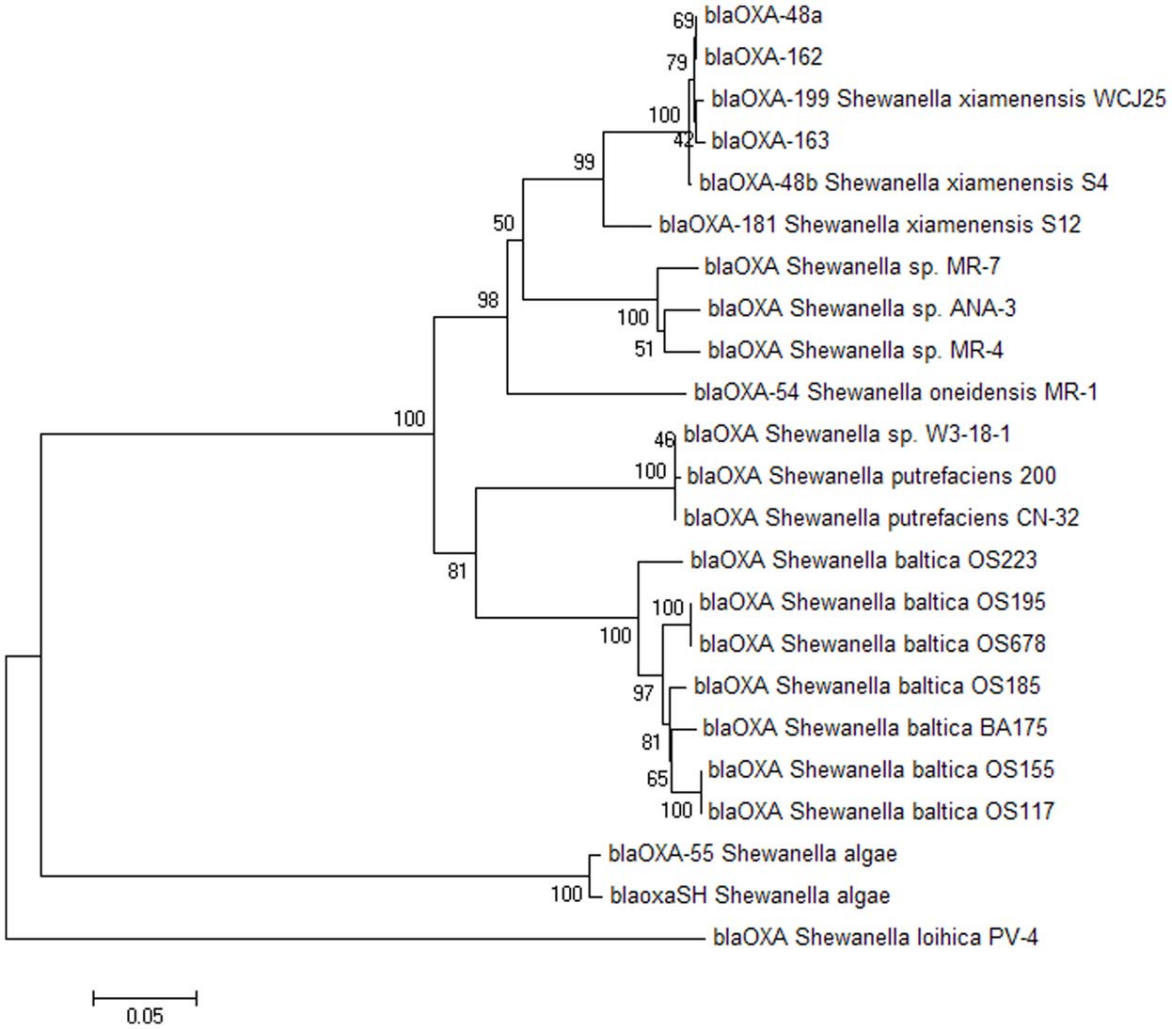
Neighbour-joining tree of *bla*_OXA-48_-like genes. Constructed using the MEGA 4.0 program with bootstrap values and the bar of distance indicated. The host species and strains for the chromosome-encoded genes are indicated. Of note, *bla*_OXA-181_ has also been found in the *Enterobacteriaceae* [10]. It appears that *bla*_OXA-48a_, *bla*_OXA-162_ and *bla*_OXA-163_ have the *S. xiamenensis* origin.

Sequences of *bla*_OXA-48_-like genes were retrieved from GenBank. The *bla*_OXA-48_-like genes and their accession numbers are *bla*_OXA-48_ (AY236073), *bla*_OXA-54_ (AY500173), *bla*_OXA-55_ (AY343493), *bla*_OXA-162_ (GU197550), *bla*_OXA-163_ (HQ700343), *bla*_OXA-181_ (JN205800) and those without assigned gene names on chromosomes of *Shewanella* spp., i.e., *S. algae* oxaSH (AY066004), *S. baltica* BA175 (CP002767), *S. baltica* OS117 (CP002811), *S. baltica* OS155 (CP000563), *S. baltica* OS185 (CP000753), *S. baltica* OS195 (CP000891), *S. baltica* OS223 (CP001252), *S. baltica* OS678 (CP002383), *S. loihica* PV-4 (CP000606), *S. oneidensis* MR-1 (AE014299), *S. putrefaciens* CN-32 (CP000681), *S. putrefaciens* 200 (CP000681), *Shewanella* sp. ANA-3 (CP000469), *Shewanella* sp. MR-4 (CP000446), *Shewanella* sp. MR-7 (CP000444) and *Shewanella* sp. W3-18-1 (CP000563). A phylogenetic tree of the *bla*_OXA-48_-like genes was constructed using the MEGA 4.0 program [9] using the neighbour-joining method and bootstrapping (value 100) (Figure 1).

### Study on Genetic Context

**Figure 2:**
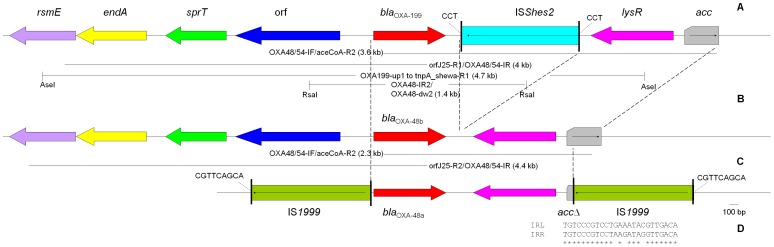
Genetic contexts of *bla*_OXA-199_ and *bla*_OXA-48_. The orientations of insertion sequences are indicated using arrows and the IRs are depicted as poles. Amplicons and sizes for PCR mapping are shown. Panel A, the genetic context of *bla*_OXA-199_. Restriction sites of enzymes that were used to generate DNA fragments as templates for inverse PCR are indicated. Common structures in the contexts of *bla*_OXA-199_, *bla*_OXA-48b_ and *bla*_OXA-48a_ are illustrated by broken lines. IS*Shes2* is inserted between *bla*_OXA-199_ and *lysR*, generating 3-bp DR (CCT). The acc gene was only partially sequenced. The gene encoding peptidase C15 is indicated as ‘orf’. Panel B, the genetic context of *bla*_OXA-48b_ in *S. xiamenensis* strain S4. Panel C, the genetic context of *bla*_OXA-48a_ in *K. pneumoniae* strain 11978 (AY236073). Two copies of IS*1999* formed a composite transposon and was inserted into the *tir* gene (responsible for transfer inhibition), which is part of the IncFII plasmid backbone, generating 9-bp DR (CGTTCAGCA). Panel D, the alignment of right-hand IR (IRR) and left-hand IR (IRL) of IS*Shes2*.

The genetic context study of *bla*_OXA-199_ was investigated using inverse PCR. Genomic DNA of WCJ25, prepared using a commercial kit (Tiangen, Beijing, China), was restricted with AseI- or RsaI (Figure 2), self-ligated with T4 DNA ligase (New England Biolabs, Ipswich, NY, USA) and then used as a template for inverse PCR. The links between genetic elements were confirmed by overlapping PCR (Figure 2, primers listed Table 1). The genetic context of *bla*_OXA-48_ in the strain S4 was characterized by PCR mapping using that of *bla*_OXA-199_ as the template (Figure 2). Primers were designed based on available sequences using the primer3 software (http://frodo.wi.mit.edu/primer3/) with the default settings. Inverse PCR, overlapping PCR and PCR mapping were also conducted using the ExTaq mix with the conditions being 94°C for 5 min, 30 cycles (94°C for 30s, 55°C for 45 s, 72°C for 5 min) and a final elongation step at 72°C for 7 min.

Amplicons were sequenced using an ABI 3730xl DNA Analyzer (Applied Biosystems, Foster City, CA) at the Beijing Genomics Institute (Beijing, China). Sequences were assembled using the SeqMan II program in the Lasergene package (DNASTAR Inc, Madison, WI) and similarity searches were carried out using BLAST programs (http://www.ncbi.nlm.nih.gov/BLAST/).

#### GenBank accession number

The genetic context of *bla*_OXA-199_ in WCJ25 and that of *bla*_OXA-48_ in the strain S4 have been deposited in GenBank as JN704570 and JX644945, respectively.

## Results and Discussion

*S. xiamenensis* is a newly-recognized species originally found in the coastal sea sediment in Xiamen, China [8] and has also been recovered from gutters in India very recently [10]. The identification of *S. xiamenensis* in India and two distant parts of China suggested that this species might be an underrecognized member of *Shewanella* with a wide geographical distribution.

The *bla*_OXA-48_-like gene of strain S4 was confirmed as a variant of *bla*_OXA-48_, designated *bla*_OXA-48b_ here, which had four silent nucleotide differences from the *bla*_OXA-48_ variant (AY236073), designated *bla*_OXA-48a_ here, found in the *Enterobacteriaceae*. Strain WCJ25 harboured a novel *bla*_OXA-48_-like gene, designated *bla*_OXA-199_ by the β-lactamases numbering system available at www.lahey.org. The *bla*_OXA-199_ gene had five nucleotide differences from both *bla*_OXA-48a_ and *bla*_OXA-48b_ (99.4% identity), specifying the OXA-199 protein with three amino acid substitutions (H37Y, V44A and D153G) compared to OXA-48. During the process of this work, *bla*_OXA-181_ was identified in a *S. xiamenensis* isolate from India [10]. However, *bla*_OXA-181_ was significantly divergent from *bla*_OXA-48b_ (94.7% identity, 42 nucleotide differences), *bla*_OXA-48a_ (94.4% identity, 45 nucleotide differences) and *bla*_OXA-199_ (94.1% identity, 47 nucleotide differences). Based on a phylogenetic tree (Figure 1) constructed by the MEGA program, the results showed that the *bla*_OXA-48_-like genes could be divided into three clusters among which *bla*_OXA-48a_, _-48b_, _-162_, _-163_, _-181_ and _-199_ were of a cluster different from the chromosome-encoded *bla*_OXA-48_-like genes of *Shewanella* species other than *S. xiamenensis*. The *bla*_OXA-48_-like genes of the same *Shewanella* species clustered together, suggesting that the divergence of the *bla*_OXA-48_-like gene might reflect the phylogeny of *Shewanella* species.

**Figure 3:**
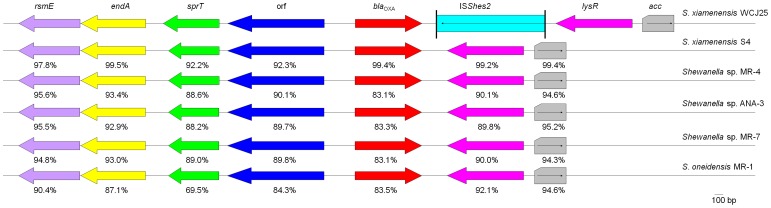
Genetic components surrounding *bla*_OXA-48_-like genes in *Shewanella* strains WCJ25, S4, MR-1, MR-4 MR-7 and ANA-3. Variants of the same gene are depicted in the same colour with nucleotide identities compared to the counterparts of WCJ25 being indicated underneath. Of note, the *acc* genes of strains MR-1, MR-4 MR-7 and ANA-3 are complete with 4554 bp in length but only 333 bp were included into the analysis in parallel with the available partial *acc* sequence of strains WCJ25 and S4.

Genetic contexts of *bla*_OXA-48b_ and *bla*_OXA-199_ were shown in Figure 2. The 26 bp sequence upstream and 63 bp downstream of *bla*_OXA-199_ were identical to those of *bla*_OXA-48a_ and *bla*_OXA-48b_, also suggesting a common origin of these genes. Both *bla*_OXA-48b_ and *bla*_OXA-199_ genes were adjacent to several genes upstream, i.e. an orf encoding the peptidase C15, *sprT* encoding a SprT-like protein of unknown function, *endA* encoding the endonuclease I and *rsmE* encoding a ribosomal RNA small subunit methyltransferase. Variants of these genes are also present adjacent to the *bla*_OXA-48_-like gene in *S. oneidensis* MR-1 (AE014299), *Shewanella* sp. MR-4 (CP000446), *Shewanella* sp. MR-7 (CP000444) and *Shewanella* sp. ANA-3 (CP000469). The nucleotide identities of these genes among *Shewanella* species are shown in Figure 3. The insertion sequence IS*Ecp1* has been found upstream of *bla*_OXA-181_ [5] but was not detected upstream of *bla*_OXA-48b_ and *bla*_OXA-199_ using long-range PCR.

As seen in the contexts of *bla*_OXA-48a_ in *K. pneumoniae* strain 11978 (AY236073) [1], a putative *lysR* transcriptional regulator gene was located downstream of *bla*_OXA-48b_ and *bla*_OXA-199_. The *lysR* gene was adjacent to an *acc* gene that encoded an acetyl-CoA carboxylase multifunctional enzyme at the other side. In *K. pneumoniae* 11978, the *acc* gene is truncated by the insertion of IS*1999* (an insertion sequence also called IS*10A*) and two copies of IS*1999* bracketing *bla*_OXA-48a_-*lysR*-*accΔ* formed a composite transposon, which could mobilize *bla*_OXA-48a_ to different locations [1], [11]. The *lysR* and *acc* genes are commonly present downstream of *bla*_OXA-48_-like genes on chromosomes of *Shewanella* spp (Figure 3). As mentioned above, genes located either upstream or downstream of the *bla*_OXA-48_-like genes from different *Shewanella* spp. displayed variable degrees of identities, suggesting that these genes might have different mutation rates.

An insertion sequence was inserted between *bla*_OXA-199_ and *lysR*, evidenced by the presence of 3 bp direct target repeats (DR) (Figure 2). This 1299-bp IS was 98.1% identical to IS*Shes2* of the IS*3* family in nucleotide sequences and had 25-bp inverted repeat sequences (IR) with 23 bp perfectly matched (Figure 2). The IS*Shes2* element has also been seen in several *Shewanella* strains whose complete genome sequences are available at GenBank, including *Shewanella* sp. MR-4 (7 copies; CP000446), *Shewanella* sp. MR-7 (9 copies plus a truncated version; CP000444), *Shewanella* sp. ANA-3 (4 copies; CP000469), *S. baltica* OS195 (2 copies; CP000891), *S. baltica* OS678 (2 copies; CP002383) and *S. baltica* OS185 (1 copy; CP000753). Other *Shewanella* strains with complete genome sequences released, including *S. baltica* OS223 (CP001252), *S. baltica* BA175 (CP002767), *S. baltica* OS117 (CP002811), *S. baltica* OS155 (CP000563), *S. woodyi* ATCC 51908 (CP000961), *S. oneidensis* MR-1 (AE014299) and *S. pealeana* ATCC 700345 (CP000851) did not harbour IS*Shes2* but instead carried other insertion sequences sharing 65.7 to 85.9% nucleotide identity with IS*Shes2*.

Based on the significant similarity among contexts of *bla*_OXA-48a_, *bla*_OXA-48b_ and *bla*_OXA-199_, it is reasonable to hypothesize that two copies of IS*1999*, one inserted at 26 bp upstream of a *bla*_OXA-48_-like gene and another inserted in *acc*, could move *bla*_OXA-48_-like-*lysR*-*accΔ* from the chromosome of *S. xiamenensis* to a plasmid. Such plasmid could have been transferred to *Enterobacteriaceae* later on resulting in the emergence of *bla*_OXA-48_-like genes. Of note, *bla*_OXA-48a_ and *bla*_OXA-181_ have always been found in distinct genetic contexts as *bla*_OXA-48a_ is bracketed by two copies of IS*1999* while *bla*_OXA-181_ is downstream of IS*Ecp1* [3]. In light of the distinct genetics and the significant nucleotide differences (94.4% identity) between *bla*_OXA-48a_ and *bla*_OXA-181_, it seems unlikely that the two genes derived from each other through mutations but had different origins from two *Shewanella* strains [3].

### Conclusions

From the phylogenetic analysis performed in this study, it appears that *bla*_OXA-48a_ might have originated from the *bla*_OXA_ genes such as *bla*_OXA-48b_ and *bla*_OXA-199_ on the chromosome of certain *S. xiamenensis* strains. The significant nucleotide differences (<95% identity) between *bla*_OXA-181_ and *bla*_OXA-48b_ or *bla*_OXA-199_ might represent the divergence of the chromosome-encoded *bla*_OXA-48_-like genes between different *S. xiamenensis* strains in different geographical regions and could also suggest that *bla*_OXA-48a_ and *bla*_OXA-181_ were mobilized independently from different *S. xiamenensis* strains. The *bla*_OXA-48a_ and *bla*_OXA-181_ determinants appeared to have distinct origins and the emergence of *bla*_OXA-48_-like genes in *Enterobacteriaceae* thus probably can not be attributed to a single mobilization event in the species *S. xiamenensis* but likely is a result of parallel or successive events occurring in multiple strains of *S. xiamenensis*.
